# Exploring the mechanisms of Guizhifuling pills in the treatment of coronary spastic angina based on network pharmacology combined with molecular docking

**DOI:** 10.1097/MD.0000000000039014

**Published:** 2024-07-19

**Authors:** Shuaimin Xu, Weiqi Cui, Xiangyu Zhang, Weijuan Song, Yanhong Wang, Yang Zhao

**Affiliations:** a Department of Pharmacy, The Fifth Affiliated Hospital of Zhengzhou University, Zhengzhou, China.

**Keywords:** coronary spastic angina, Guizhifuling pills, molecular docking, network pharmacology

## Abstract

Coronary spastic angina (CSA) is common, and treatment options for refractory vasospastic angina are sometimes limited. Guizhifuling pills (GFP) have demonstrated efficacy in reducing CSA episodes, but their pharmacological mechanism remains unclear. To explore the mechanism of action of GFP in preventing and treating CSA, we employed network pharmacology and molecular docking to predict targets and analyze networks. We searched GFP chemical composition information and related targets from databases. The drug-target and drug-target pathway networks were constructed using Cytoscape. Then the protein–protein interaction was analyzed using the STRING database. Gene Ontology biological functions and Kyoto Encyclopedia of Genes and Genomes pathways were performed by the Metascape database, and molecular docking validation of vital active ingredients and action targets of GFP was performed using AutoDock Vina software. The 51 active components in GFP are expected to influence CSA by controlling 279 target genes and 151 signaling pathways. Among them, 6 core components, such as quercetin, β-sitosterol, and baicalein, may regulate CSA by affecting 10 key target genes such as *STAT3*, *IL-6, TP53*, *AKT1*, and *EGFR*. In addition, they are involved in various critical signaling pathways such as apelin, calcium, advanced glycation end product–receptor for advanced glycation end product, and necroptosis. Molecular docking analysis confirms favorable binding interactions between the active components of GFP and the selected target proteins. The effects of GFP in treating CSA involve multiple components, targets, and pathways, offering a theoretical basis for its clinical use and enhancing our understanding of how it works.

## 1. Introduction

Coronary spastic angina (CSA) is a condition caused by coronary artery spasm (CAS) resulting in angina pectoris and may also lead to myocardial infarction (MI), ventricular arrhythmias, and sudden death.^[1,2]^ Prinzmetal et al^[3]^ described a syndrome of nonexertional chest pain with ST-segment elevation on electrocardiography in 1959. This syndrome became known as Prinzmetal or variant angina and was thought to be due to vasospasm in coronary arteries without obstructive lesions. In many cases, CAS can occur spontaneously without an identifiable cause. Known triggers of spasm in susceptible patients include hyperventilation, cocaine use, smoking, and the administration of provocative agents such as acetylcholine, ergonovine, histamine, sumatriptan, or serotonin.^[4]^ Currently, the pathophysiological mechanisms leading to coronary vasospasm are not fully understood. While some studies have specifically implicated nitric oxide (NO) deficiency, it is postulated to play a crucial role in the development of coronary vasospasm.^[5]^ Alternative mechanisms of coronary vasospasm include increased phospholipase C activity.^[6]^ Furthermore, coronary vasospasm is associated with elevated markers of oxidative stress and inflammation, including thioredoxin, C-reactive protein, and monocyte levels.^[7]^ Certain behavioral traits, such as type A personality, panic disorder, and severe anxiety, have also been described as being associated with coronary vasospasm.^[8]^

Calcium channel blockers (CCBs) are the primary treatment approach for CSA.^[9]^ However, 10% to 20% of patients with CSA are refractory to or intolerant of CCBs. Furthermore, long-acting nitrates can be used in combination with CCBs when the latter are ineffective.^[10]^ Other agents, such as endothelin antagonists, such as bosentan^[11]^ and rho-kinase inhibitors, such as fasudil,^[12]^ have been investigated with varying degrees of success. Preliminary studies on cilostazol have shown promising results, but remain limited^[13]^; further research is needed to validate its clinical application. Traditional Chinese medicine has a long history of use in the treatment of coronary heart disease, and Guizhifuling pills (GFP) are one of the commonly prescribed herbal formulations.^[14]^ GFP is derived from “Jin Gui Yao Lue,” the earliest existing Chinese monograph on the diagnosis and treatment of various diseases. GFP is composed of Guizhi (GZ), Fuling (FL), Mudanpi (MDP), Chishao (CS), and Taoren (TR), and it exhibits a diverse range of pharmacological effects, including improving blood circulation, inhibiting platelet aggregation, and having anti-inflammatory properties.^[15,16]^ However, the precise mechanism by which GFP exerts its therapeutic effects in the management of CSA remains unclear.

**Figure 1.**
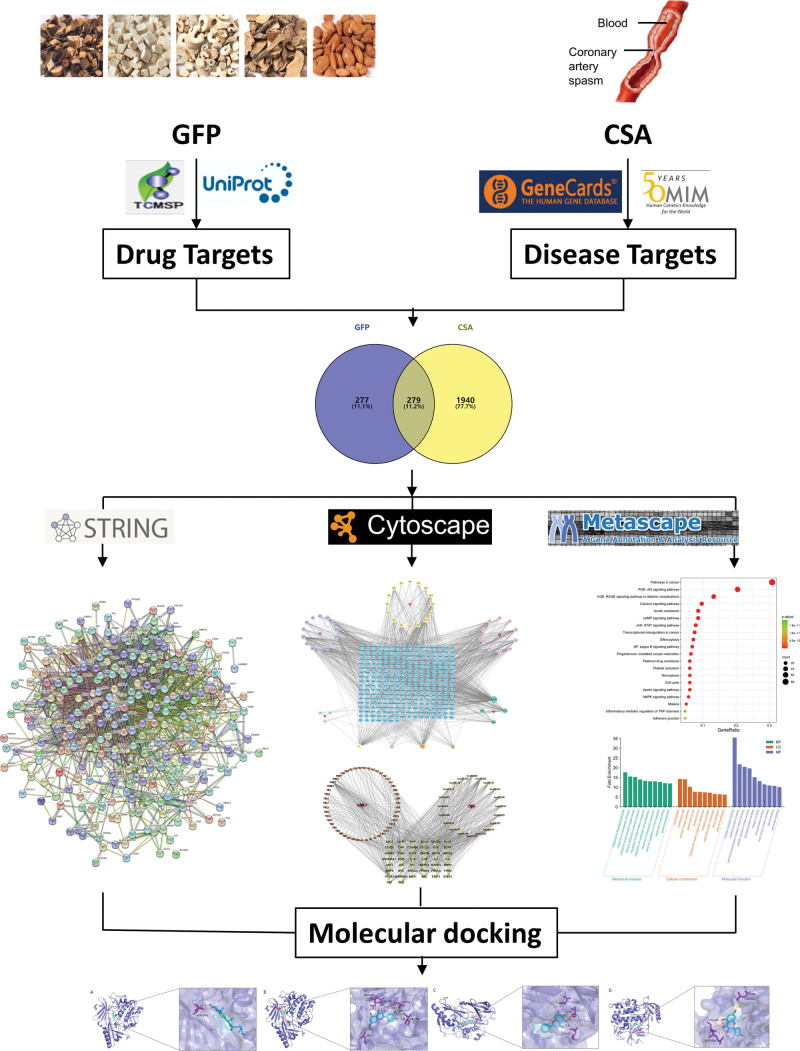
The network pharmacological study of GFP for the treatment of CSA diagram. CSA = coronary spastic angina, GFP = Guizhifuling pills.

The complex composition and multiple targets of traditional Chinese medicine pose challenges for studying clinical diseases due to their vague and unclear mechanisms of action. However, network pharmacology has emerged in response to rapid advancements in systems biology and computer technology. It utilizes the interaction network of drug disease to systematically analyze the interventions and impacts of drugs on disease networks, shedding light on the enigmatic synergistic effects of multimolecular drugs within the human body.^[17]^ Molecular docking is a theoretical simulation method used to investigate the interaction between small molecule ligands and protein receptors, predicting their binding modes and affinities. Therefore, this study utilized network pharmacology to predict the chemical composition of GFP, identify potential targets and key pathways involved in its treatment of CSA, and employed molecular docking technology to simulate the binding activity between active components and core targets, aiming to provide a theoretical foundation for elucidating the mechanism of GFP in CSA treatment. The detailed flowchart is depicted in Figure 1.

## 2. Methods

### 2.1. Identification of the active components and targets of GFP

GFP consists of 5 herbs, namely GZ, FL, MDP, CS, and TR. The constituents of these herbs were researched and collected from the Traditional Chinese Medicine Systems Pharmacology Database and Analysis Platform (https://tcmsp-e.com/). The relevant compounds and their corresponding target proteins were then retrieved using the screening criteria of oral bioavailability ≥ 30% and drug-likeness ≥ 0.18. The UniProt database (https://www.uniprot.org/) was utilized for target protein names normalization, with species limited to “Homo sapiens.”

### 2.2. Establishment of CSA-related target genes database

The search term “coronary spastic angina” was utilized to identify disease targets in GeneCards (https://www.genecards.org/) and OMIM (https://www.omim.org/). The target genes associated with CSA were subsequently obtained by removing duplicate entries.

### 2.3. Screening the common target genes of GFD and CSA

The target genes of GFP and CSA were analyzed using Venny 2.1 (https://bioinfogp.cnb.csic.es/tools/venny/) to obtain the intersection target genes, which are the potential target genes of GFP for CSA.

### 2.4. Constructing the drug-component-potential target gene-disease network

The screened target genes of the 5 herbal medicines (GZ, FL, MDP, CS, and TR), the intersection target genes, and CSA were used as targets. The Cytoscape 3.9.1 software was used to construct a “drug-component-potential target gene-disease” network.

### 2.5. Construction and analysis of protein–protein interaction network

The intersection targets of GFP and CSA were uploaded to the STRING database (https://cn.string-db.org/), with the species condition set to “Homo sapiens” and a minimum required interaction score of 0.7. In addition, free nodes were removed. The obtained results were then imported into Cytoscape 3.9.1 for visualization.

### 2.6. Gene Ontology and Kyoto Gene and Genome Encyclopedia pathway enrichment analysis

The common targets of GFP and CSA were submitted to Metascape (https://www.metascape.org/) for enrichment analysis of Gene Ontology (GO) and the Kyoto Encyclopedia of Genes and Genomes (KEGG). The species was specified as “H. sapiens,” with a minimum overlap value of 3 and significance level set at *P* < .01. GO analysis individually considers the top 10 results for biological process (BP), cellular component (CC), and molecular function (MF), and KEGG analysis are sorted according to the *P*-value. All results were plotted by Bioinformatics (https://www.bioinformatics.com.cn/) as a 3-in-1 histogram of BP, CC, and MF, and a KEGG bubble diagram.

### 2.7. Molecular docking

The primary active compounds of GFP were converted into SDF format files representing their 2-dimensional structures using the PubChem database, then optimized in Chem3D and saved in mol2 format. Crystal structures of the core target proteins were obtained from the PDB database (https://www.rcsb.org/). For the analysis of binding affinities and interaction modes between the drug candidate and its targets, we used AutoDock Vina 1.1.2, a computational protein-ligand docking software. For molecular docking, 6 primary compounds and 10 core targets were selected, with 20 binding energy sites retained for each docking pair. The conformation with the highest affinity was selected as the final docked conformation. The docking results were analyzed and visualized using PyMOL Version 2.4.0a0 Open-Source for the top 5 models with the lowest binding energies.

### 2.8. Statistical analysis

All statistical analyses and visualization were performed using Cytoscape 3.9.1, AutoDock Vina 1.1.2, and PyMOL Version 2.4.0a0 Open-Source.

## 3. Results

### 3.1. Active ingredients and target genes database of GFP

The components of GFP were searched through the Traditional Chinese Medicine Systems Pharmacology Database and Analysis Platform, and 51 active ingredients were identified, as shown in Table 1 and Table S1, Supplemental Digital Content, http://links.lww.com/MD/N244. The number of active ingredients was 17 for CS, 11 for FL, 6 GZ, 6 for MDP, and 18 for TR, respectively. Among the herbal components, CS, GZ, and MDP share 2 common components (sitosterol and (+)-catechin); CS, GZ, and TR share a common component (beta-sitosterol); and FL and TR share a common component (hederagenin). After removing duplicates of target genes corresponding to the 85 active ingredients, a total of 556 unique drug targets were identified.

**Table 1:** The active ingredients of Guizhifuling pills (only the top 10 compounds of DL are shown, the rest are shown in Supplementary Table S1, Supplemental Digital Content, http://links.lww.com/MD/N244).

Mol ID	Molecule name	Code name	OB (%)	DL	Drug
MOL000273	(2R)-2-[(3S,5R,10S,13R,14R,16R,17R)-3,16-dihydroxy-4,4,10,13,14-pentamethyl-2,3,5,6,12,15,16,17-octahydro-1H-cyclopenta[a]phenanthren-17-yl]-6-methylhept-5-enoic acid	FL1	30.93	0.81	FL
MOL000283	Ergosterol peroxide	FL5	40.36	0.81	FL
MOL000287	3beta-hydroxy-24-methylene-8-lanostene-21-oic acid	FL6	38.70	0.81	FL
MOL000289	pachymic acid	FL7	33.63	0.81	FL
MOL001921	Lactiflorin	CS3	49.12	0.80	CS
MOL000275	Trametenolic acid	FL2	38.71	0.80	FL
MOL001924	Paeoniflorin	CS4	53.87	0.79	CS
MOL000211	Mairin	MDP1	55.38	0.78	MDP
MOL001323	Sitosterol alpha1	TR1	43.28	0.78	TR
MOL007004	Albiflorin	CS14	30.25	0.77	CS

CS = Chishao, DL = drug-likeness, FL = Fuling, MDP = Mudanpi, OB = oral bioavailability, TR = Taoren.

### 3.2. CSA-related target genes

The search term “coronary spastic angina” was used, and 1869 and 378 target genes were obtained from databases GeneCards and OMIM, respectively. After merging these 3 databases and removing duplicates, a total of 2219 unique target genes were obtained.

### 3.3. Screening the common targets of GFP and CSA

**Figure 2.**
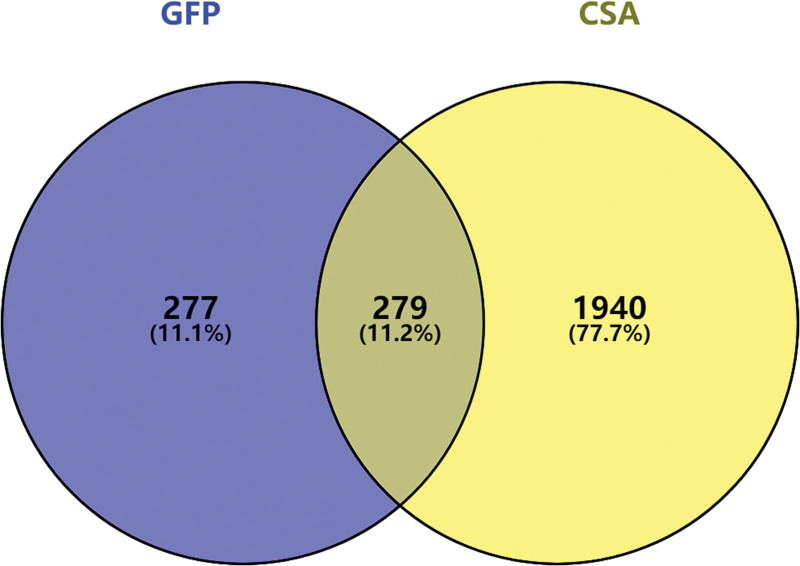
The Venn diagram of GFP and CSA intersection targets. CSA = coronary spastic angina, GFP = Guizhifuling pills.

The 556 drug targets and 2219 disease targets were input into Venny 2.1 to draw a Venn diagram. The overlap between the 2 sets resulted in 279 common targets, representing potential targets for treating CSA with GFP, as shown in Figure 2.

### 3.4. Construction and analysis of drug–target network

**Figure 3.**
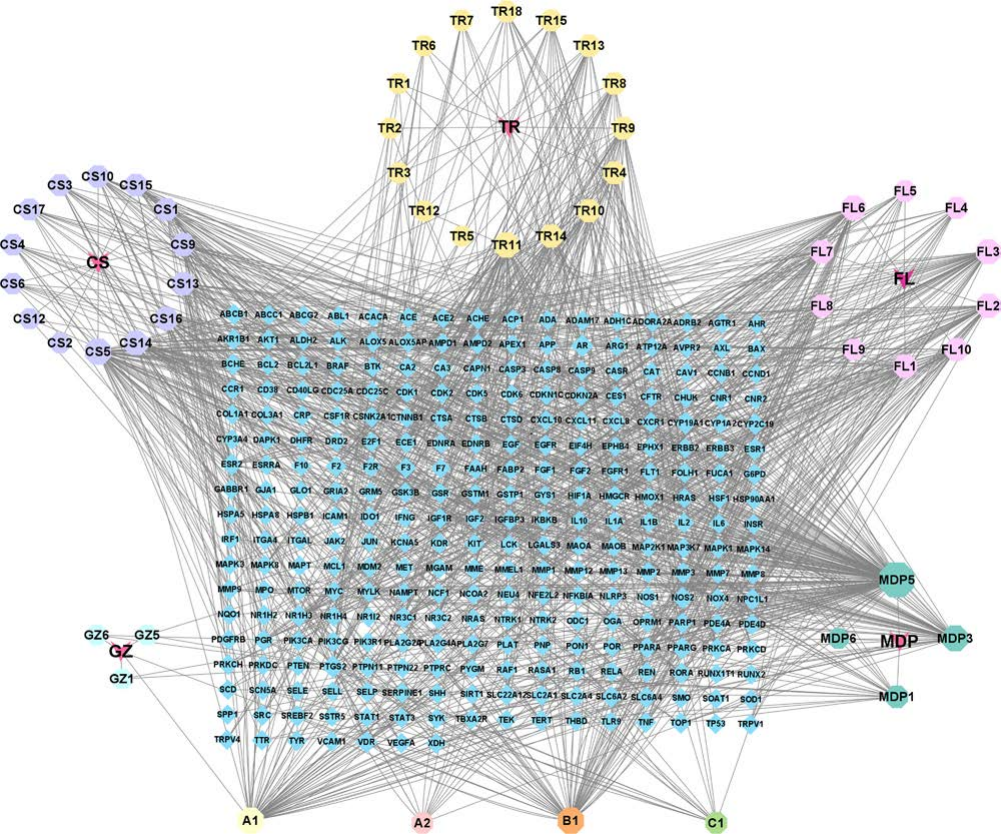
Drug-target network diagram. Blue represents the common targets; red represents drugs; other colors represent the active ingredient of each drug. CS = Chishao, FL = Fuling, GZ = Guizhi, MDP = Mudanpi, TR = Taoren.

The drug-target network was constructed using Cytoscape 3.9.1 software to represent the intricate relationship between GFP components and their potential role in the treatment of CSA, as shown in Figure 3. The network contains a total of 335 nodes and 1270 edges, representing the complex interactions between different drug components, targets, and disease. Higher degree nodes are considered to be more central and potentially more influential within the network. After reviewing the literature on the 10 components with the highest degrees, we selected 6 compounds, such as quercetin, beta-sitosterol, baicalein, kaempferol, cerevisterol, and albiflorin, that showed greater relevance to the treatment of CSA for further investigation through molecular docking studies. These results provide valuable insights into exploring the potential of GFP for the treatment of CSA.

### 3.5. Construction and analysis of core targets protein–protein interaction network

**Figure 4.**
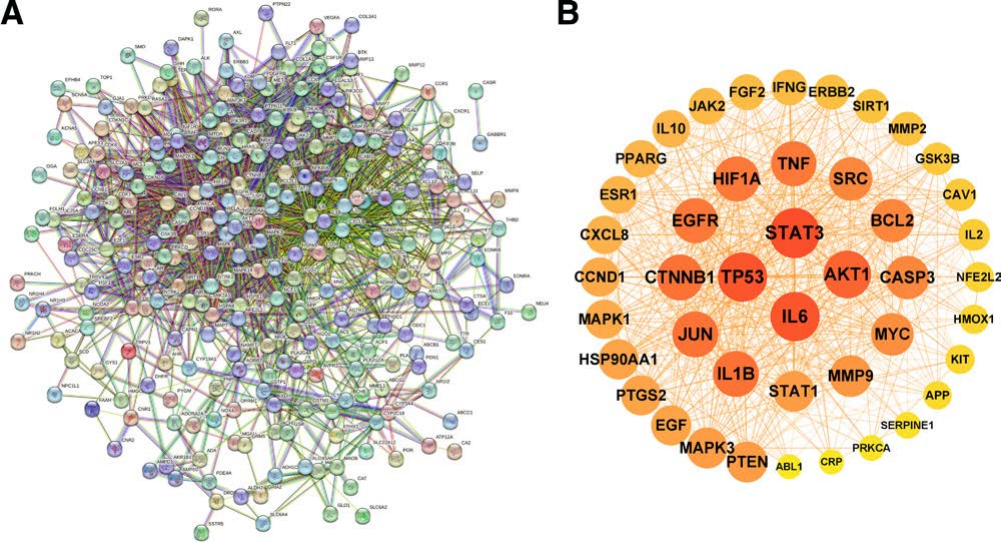
The target genes and PPI network map of GFP therapy for CSA. (A) The PPI network of common target genes. (B) The PPI network of core target genes. CSA = coronary spastic angina, PPI = protein–protein interaction.

The protein–protein interaction (PPI) network mapped by introducing 279 common target genes into the STRING online platform is shown in Figure 4A. Import the “tsv” file obtained from the STRING online platform analysis into the Cytoscape 3.9.1 software, and the function “centiscape2.2” was used to find the core targets. A total of 44 core target genes were screened by constraining Closeness unDir to exceed 0.001459189, Betweenness unDir to exceed 440.3731343, and Degree unDir to exceed 18.92537313, and their PPI network is shown in Figure 4B, among which node size from large to small and color from dark to light represent a gradual decrease in the degree value from large to small. The specific information for the core targets is shown in Table 2 and Table S2, Supplemental Digital Content, http://links.lww.com/MD/N245.

**Table 2:** Specific information for core targets (only the top 10 genes of degree are shown, the rest are shown in Supplementary Table S2, Supplemental Digital Content, http://links.lww.com/MD/N245).

Number	Uniprot ID	Gene name	Protein name	Degree
1	P40763	*STAT3*	Signal transducer and activator of transcription 3	39
2	P05231	*IL-6*	Interleukin-6	38
3	P04637	*TP53*	Cellular tumor antigen p53	38
4	P31749	*AKT1*	RAC-alpha serine/threonine-protein kinase	36
5	P01584	*IL1B*	Interleukin-1 beta	34
6	P35222	*CTNNB1*	Catenin beta-1	34
7	P05412	*JUN*	Transcription factor Jun	34
8	Q16665	*HIF1A*	Hypoxia-inducible factor 1-alpha	33
9	P01375	*TNF*	Tumor necrosis factor	33
10	P00533	*EGFR*	Epidermal growth factor receptor	33

### 3.6. GO and KEGG pathway enrichment analysis for targets

**Figure 5.**
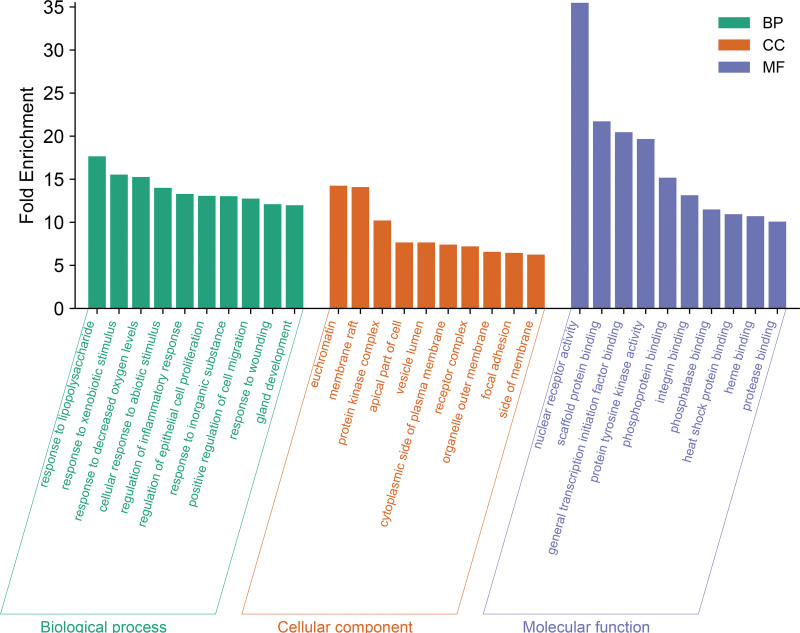
The 3-in-1 histogram of BP, CC, and MF. BP = biological process, CC = cellular component, MF = molecular function.

A total of 2694 BPs, 134 CCs, and 262 MFs were identified from 279 common targets through GO enrichment analysis, using a significance threshold of *P* < .01. The bioinformatics was used to visualize the selection of the top 10 entries with the highest fold enrichment values, as shown in Figure 5. BPs included responses to lipopolysaccharide, xenobiotic stimuli, decreased oxygen levels, abiotic stimuli, and the regulation of inflammatory responses, among others. CC included comprised euchromatin, membrane rafts, protein kinase complexes, the apical part of cells, vesicle lumens, and others. MFs included nuclear receptor activity, scaffold protein binding, binding to general transcription initiation factors, protein tyrosine kinase activity, phosphoprotein binding, and more.

**Figure 6.**
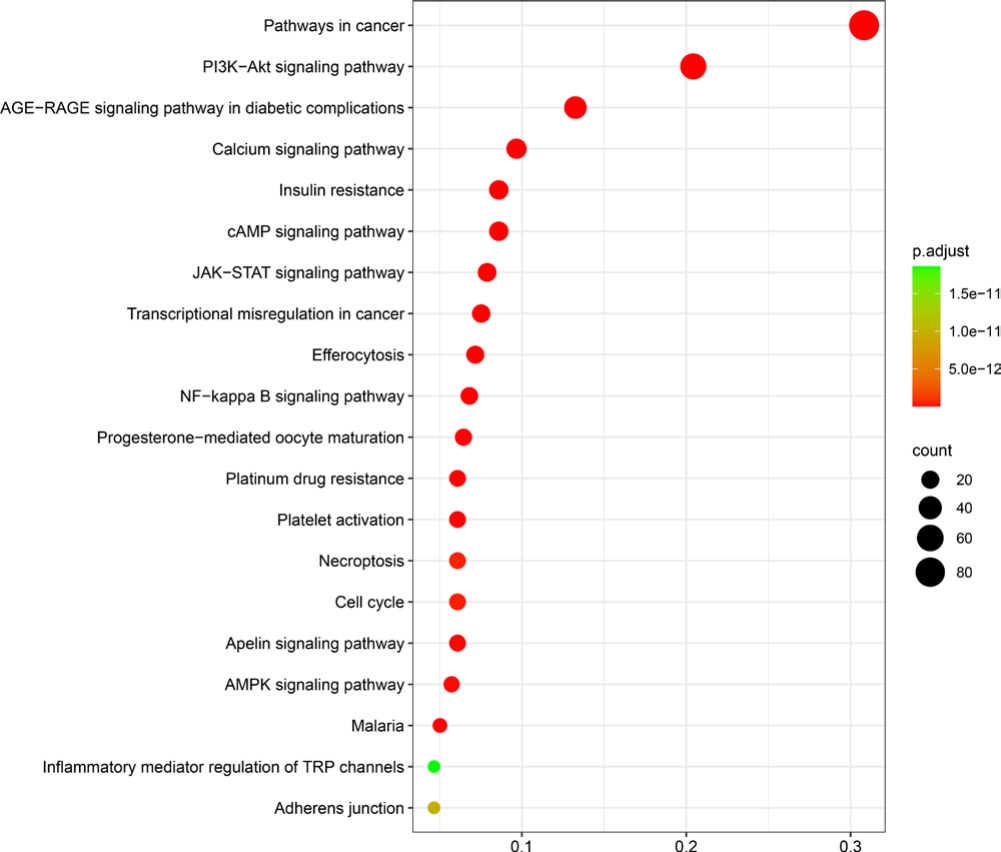
Bubble chart of the top 20 significantly enriched terms in Kyoto Encyclopedia of Genes and Genomes pathways. The color coding indicates the significance (adjusted *P*-value) of each pathway, with red indicating highly significant and green and yellow indicating less significant. AGE–RAGE = advanced glycation end products–receptor for advanced glycation end product.

**Figure 7.**
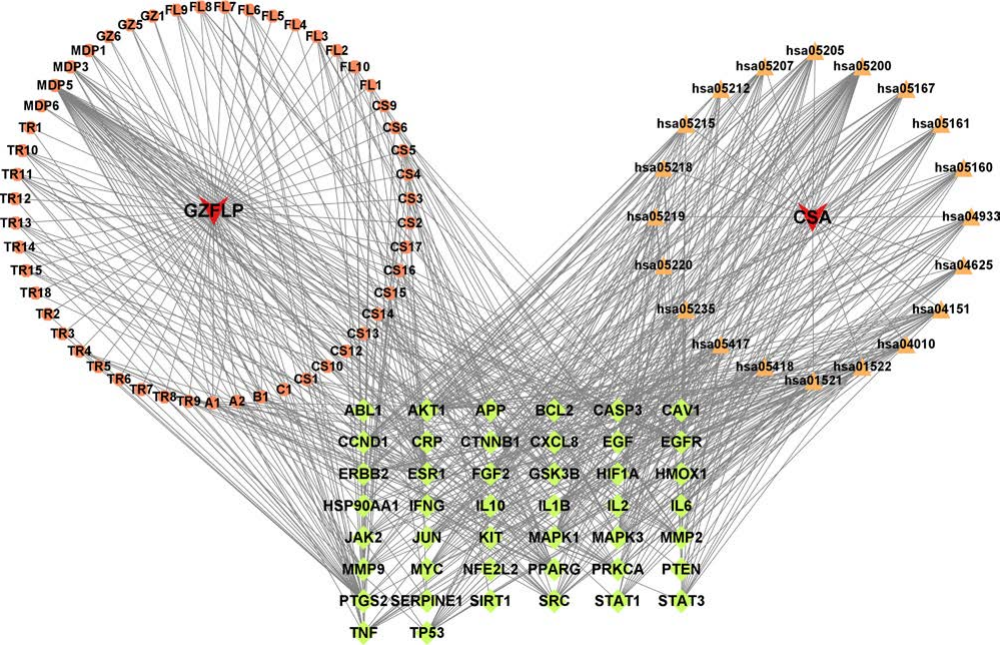
The drug-target-pathway network. Brown indicates drug active ingredients; orange indicates the pathway; green indicates the core targets. CS = Chishao, CSA = coronary spastic angina, FL = Fuling, GZ = Guizhi, MDP = Mudanpi, TR = Taoren.

KEGG pathway enrichment analysis identified 151 significantly enriched pathways (*P* < .01) that are potentially targeted by the active components of GFP in the treatment of CSA. The lower the *P*-value, the more significant the enrichment. The top 20 pathways (Table S3, Supplemental Digital Content, http://links.lww.com/MD/N246), ranked by *P*-value, were visualized using a bioinformatics platform, as shown in Figure 6. The top-ranked pathways included Pathways in cancer, PI3K-Akt signaling pathway, advanced glycation end products (AGE)–receptor for advanced glycation end product (RAGE) signaling pathway in diabetic complications, Insulin resistance, and Calcium signaling pathway. These findings suggest that the active components of GFP primarily target these pathways in the treatment of CSA. The top 20 KEGG pathways, 44 core targets, and 51 active ingredients were visualized using Cytoscape 3.9.1 to map the drug-target-pathway network, as shown in Figure 7.

### 3.7. Molecular docking results

**Figure 8.**
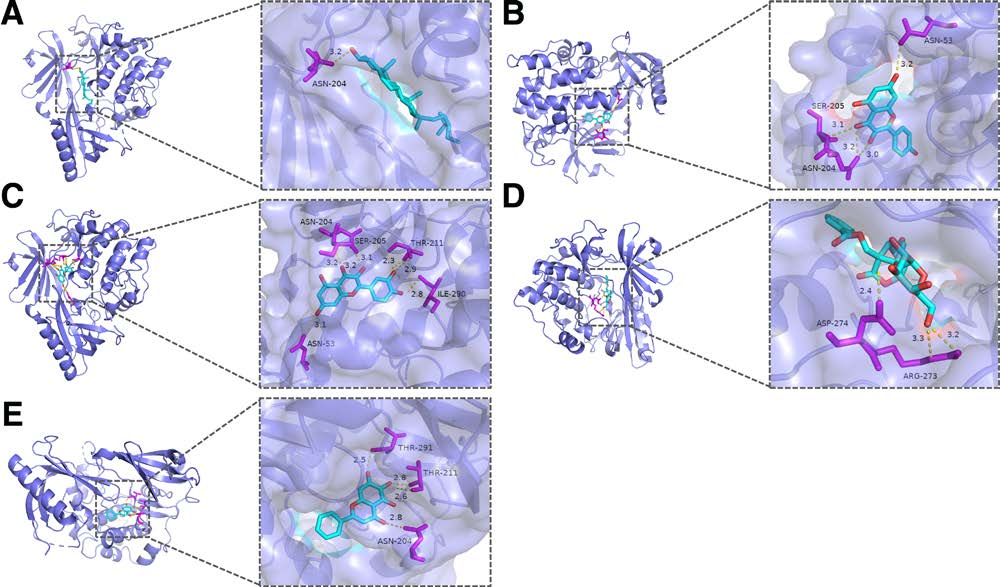
The top 5 significant molecular docking. (A) The docking diagram of AKT1 and beta-sitosterol (binding energy = −11.0 kcal/mol). (B) The docking diagram of AKT1 and kaempferol (binding energy = −9.8 kcal/mol). (C) The docking diagram of ATK1 and quercetin (binding energy = −9.6 kcal/mol). (D) The docking diagram of AKT1 and albiflorin (binding energy = −9.6 kcal/mol). (E) The docking diagram of AKT1 and baicalein (binding energy = −9.5 kcal/mol). AKT1 = RAC-alpha serine/threonine-protein kinase.

The top 10 core target genes of the PPI network were verified by molecular docking with the top 6 core components of the drug-component-target-disease network. These core target genes include STAT3 (PDB ID: 6njs), IL-6 (PDB ID: 1alu), TP53 (PDB ID: 3dcy), AKT1 (PDB ID: 7nh5), IL1B (PDB ID: 4g6j), CTNNB1 (PDB ID: 1jdh), JUN (PDB ID: 5t01), HIF1A (PDB ID: 1lm8), TNF (PDB ID: 2az5), and EGFR (PDB ID: 5ug9). The molecular docking results for the 60 receptor-ligand pairs are presented in Table 3. The molecular docking results showed the following binding energies for the top 5 ligands interacting with the AKT1 target: beta-sitosterol (−11.0 kcal/mol), kaempferol (19.8 kcal/mol), quercetin (−9.6 kcal/mol), albiflorin (−9.6 kcal/mol), and baicalein (−9.5 kcal/mol). These visualization results are presented in Figure 8.

**Table 3:** Molecular docking results of key active ingredients and the core targets of Guizhifuling pills.

		Binding energy (kcal/mol)									
Mol ID	Active ingredient	STAT3	IL-6	TP53	AKT1	IL1B	CTNNB1	JUN	HIF1A	TNF	EGFR
MOL000098	Quercetin	−7.3	−7.0	−8.0	−9.6	−7.5	−7.4	−8.2	−6.9	−7.1	−8.3
MOL000358	Beta-sitosterol	−6.7	−6.8	−7.5	−11.0	−7.7	−7.0	−7.8	−7.3	−8.9	−9.1
MOL002714	Baicalein	−7.5	−6.9	−8.1	−9.5	−7.6	−6.7	−7.9	−7.1	−8.1	−8.4
MOL000422	Kaempferol	−7.8	−6.8	−8.0	−9.8	−7.4	−6.8	−7.7	−6.5	−7.1	−8.4
MOL000279	Cerevisterol	−6.9	−7.6	−8.5	−8.0	−8.2	−6.2	−8.2	−7.5	−8.4	−8.6
MOL007004	Albiflorin	−7.6	−6.8	−8.3	−9.6	−7.6	−6.5	−8.5	−7.5	−8.2	−7.1

AKT1 = RAC-alpha serine/threonine-protein kinase, CTNNB1 = catenin beta-1, EGFR = epidermal growth factor receptor, HIF1A = hypoxia-inducible factor 1-alpha, IL1B = interleukin-1 beta, IL-6 = interleukin-6, JUN = transcription factor Jun, STAT3 = signal transducer and activator of transcription 3, TNF = tumor necrosis factor, TP53 = cellular tumor antigen p53.

## 4. Discussion

CAS angina is a variant form of angina. Epidemiological studies have found that among all MI patients, 1% to 15% show nonobstructive coronary artery disease on angiography, with approximately 30% confirmed to have CAS.^[18]^ In addition, some studies have suggested a higher prevalence of the condition in males compared to females within certain populations, though this finding may be confounded by higher smoking rates among males.^[19]^ Research indicates that the mechanisms underlying CAS involve multiple factors, including endothelial dysfunction, autonomic nervous system dysregulation, disruption of the balance between endogenous vasodilators and vasoconstrictors, increased sensitivity of vascular smooth muscle cells to contraction, magnesium deficiency, oxidative stress, chronic inflammation, and psychological stress.^[4,16]^

Modern pharmacological studies have demonstrated that GFP exhibits significant anti-inflammatory, anticancer, anticoagulant, neuroregulatory, metabolic regulatory, and immunomodulatory effects.^[16,20,21]^ In this study, utilizing network pharmacology and constructing a network of Chinese herbal compound regulation, we identified 6 key component nodes with the closest associations to cardiovascular diseases among the top 10 active components ranked by degree. These components, ranked in descending order of degree value, include quercetin, beta-sitosterol, baicalein, kaempferol, cerevisterol, and albiflorin, suggesting their potential crucial roles in the treatment of CSA. Quercetin and beta-sitosterol possess the ability to inhibit inflammatory responses by reducing the release of inflammatory mediators and suppressing leukocyte activation, thereby lowering the level of inflammation in the blood vessel walls. In addition, quercetin and beta-sitosterol can promote the production of NO by vascular endothelial cells, leading to vasodilation, blood pressure reduction, improved vascular function, and prevention of cardiovascular diseases. Furthermore, quercetin and beta-sitosterol also exhibit pharmacological effects such as lowering low-density lipoprotein cholesterol, inhibiting platelet aggregation, and antioxidation.^[22,23]^ Baicalein is a natural flavonoid compound capable of scavenging free radicals and peroxides in the body, thereby reducing oxidative stress-induced damage to cells. Moreover, research suggests that baicalein exerts antihypertensive effects through mechanisms such as vasodilation and inhibition of angiotensin-converting enzyme.^[24]^ Albiflorin is a natural monoglycoside compound with multiple pharmacological effects. Studies indicate that albiflorin can relax smooth muscle, exert antispasmodic effects, and possess certain antidepressant properties, improving mood and alleviating depressive symptoms.^[25]^ Kaempferol and cerevisterol can increase insulin sensitivity, improve insulin resistance, and regulate glucose metabolism. In addition, kaempferol and cerevisterol exhibit other pharmacological effects such as anticancer activity, antioxidative stress, and anti-inflammatory effects.^[26,27]^ Indeed, it is the synergistic pharmacological actions of the aforementioned key compounds that contribute to the remarkable efficacy of GFP in the treatment of CSA. Multiple case reports have confirmed that in patients with refractory CAS angina who are refractory to standard treatment regimens, combination therapy with GFP can significantly improve symptoms and reduce the frequency of angina attacks.^[28–30]^

The PPI network analysis has identified 10 potential key targets, including STAT3, IL-6, TP53, and AKT1. STAT3 is implicated in the regulation of several physiological processes, such as cell proliferation, differentiation, survival, and immune response. The dysregulation of the STAT3 signaling pathway has been associated with the development and progression of various diseases, including cancer, autoimmune, inflammatory, and neurological disorders.^[31]^ IL-6 can promote the differentiation of B cells and activation of T cells, enhance acute inflammatory responses, and regulate the hepatic synthesis of acute-phase proteins. The disruption of the IL-6 signaling pathway has been associated with the pathogenesis of various diseases, including autoimmune diseases, cancer, cardiovascular diseases, and neurological disorders.^[32]^ AKT1 is a key cell signaling molecule that plays a crucial role in maintaining normal physiological function and preventing disease development, particularly in the processes of vascular endothelial cell proliferation, migration, survival, and cardiomyocyte survival. The dysregulation of the AKT1 signaling pathway has been associated with the pathogenesis of atherosclerosis, MI, and heart failure.^[33]^ IL1B is a key regulator of immune and inflammatory responses, as well as wound healing. Furthermore, IL1B can stimulate the activation of T and B cells, promote the production of inflammatory mediators such as PGE2 and NO, and induce acute inflammatory responses. The overexpression of IL1B can induce the apoptosis of endothelial and smooth muscle cells, exacerbate endothelial dysfunction, and promote the differentiation of smooth muscle cells into osteoblast-like cells, thereby accelerating the process of arterial calcification.^[34]^ TNF is a critical regulator of immune and inflammatory responses, as it can activate immune cells, induce the production of inflammatory factors, and promote cell apoptosis. The overexpression of TNF has been associated with the pathogenesis of various inflammatory diseases, autoimmune diseases, and cancers.^[35]^ The overexpression of these key targets can generally increase the body’s inflammatory response, and the inflammatory response is one of the main causes of endothelial dysfunction. Previous studies have suggested that the pathogenesis of CSA may be related to endothelial dysfunction, and therefore, one of the potential mechanisms of GFP treatment for CSA may be to improve endothelial function by regulating the body’s inflammatory response, thereby reducing the frequency of CSA attacks.

We performed GO enrichment analysis for 279 common targets. It was found that GFP affected the response to lipopolysaccharide, response to decreased oxygen levels regulation of inflammatory response, nuclear receptor activity, scaffold protein binding, euchromatin, membrane raft, etc, to treat CSA. Initial screening of the first 20 KEGG pathways identified the apelin signaling pathway, calcium signaling pathway, AGE–RAGE signaling pathway in diabetic complications, and necroptosis as the most likely key pathways for GFP treatment of CSA. The apelin signaling pathway is an important neuroendocrine system consisting of apelin and its receptor APJ. Apelin promotes vasodilation by activating the APJ receptor, which stimulates the production of several vasodilatory factors, such as NO and superoxide dismutase, in vascular endothelial cells.^[36]^ Studies have found that the expression of the apelin signaling pathway in patients with coronary artery disease is negatively correlated with the occurrence of CAS.^[37]^ These findings suggest that the apelin signaling pathway plays a crucial role in the onset and progression of cardiovascular diseases, particularly those associated with vasospasm. Further studies are expected to provide important insights into the pathogenesis of CSA and the development of new therapeutic strategies.

The calcium signaling pathway is an important intracellular signaling pathway. Within the cardiovascular system, Ca^2+^ signaling plays a crucial role in regulating myocardial contraction, vascular smooth muscle contraction and relaxation, as well as endothelial function. Research has shown that Ca^2+^/calmodulin-dependent protein kinase II promotes smooth muscle cell contraction by phosphorylating l-type calcium channels, myosin light chain kinase, and other targets, thereby leading to vasospasm.^[38]^ Furthermore, the calcium signaling pathway exhibits significantly increased activity in patients with CAS, a condition closely related to the occurrence and development of vasospasm.^[39]^ The AGE–RAGE signaling pathway plays a crucial role in the development and progression of cardiovascular diseases, particularly those associated with vasospasm. Studies have shown that the activity of the AGE–RAGE signaling pathway is significantly increased in patients with CAS and that the level of AGEs is positively correlated with the occurrence of CAS. Furthermore, the AGE–RAGE signaling pathway is involved in the proliferation and migration of vascular smooth muscle cells, promoting intima-media thickening and vascular stenosis, which in turn exacerbates the onset and development of vasospasm.^[40,41]^ Necroptosis is a form of regulated cell death. Research has shown that in patients with CASs, the number of necroptotic cells significantly increases, correlating with the occurrence and severity of spasms as well as cardiovascular events.^[42]^

Molecular docking studies revealed that all components of the key compounds exhibit binding energies < −5 kcal/mol with their corresponding target proteins, falling within the moderate binding energy range. This suggests the bioactivity of the screened key components. Notably, the binding energies of the selected ligands with AKT1 were generally low, with the best docking result observed with beta-sitosterol at −11 kcal/mol. Furthermore, research has shown that activation of AKT1 can promote the production of NO, enhancing the vasodilation capacity of endothelial cells.^[43]^ Moreover, Akt1 signaling is necessary for ischemic resolution postinjury and likely reflects the consequence of NO insufficiency critical for vascular repair.^[44]^ The findings of this study provide new targets and strategies for the prevention and treatment of CSA.

However, there is a lack of a unified analysis process and evaluation standard for network pharmacology research, and the methods and tools used by different researchers may vary, which affects the comparability and reliability of the results. In addition, the existing docking algorithms of molecular docking cannot completely simulate the real molecular dynamics process. The future direction of the development of network pharmacology should involve combining with other computer-aided drug design techniques, improving the docking algorithms, and integrating more experimental data to enhance the predictive ability and application scope of network pharmacology and molecular docking in new drug discovery and development.

## 5. Conclusions

This study systematically predicted, screened, and analyzed the potential targets and pathways that may play important roles in BPs, elucidating the possible mechanisms of GFP in the prevention and treatment of CSA. Most importantly, these results provide evidence and new insights for further research into the pharmacological mechanisms of GFP.

## Author contributions

**Conceptualization:** Shuaimin Xu.

**Software:** Shuaimin Xu.

**Writing—review and editing:** Shuaimin Xu.

**Data curation:** Weiqi Cui.

**Writing—original draft:** Weiqi Cui.

**Formal analysis:** Xiangyu Zhang.

**Methodology:** Weijuan Song.

**Resources:** Yanhong Wang.

**Visualization:** Yang Zhao.

## Supplementary Material






