# Supplementary material for: Exploring the mechanisms of Guizhifuling pills in the treatment of coronary spastic angina based on network pharmacology combined with molecular docking
Source: Medicine (Baltimore). 2024 Jul 19;103(29):e39014. doi: 10.1097/MD.0000000000039014 (PMC11398759; doi:10.1097/MD.0000000000039014)
Supplement: Supplementary file 1 [file medi-103-e39014-s001.docx]

**Table S1** The active ingredients of GFP.

| **Mol ID** | **Molecule Name** | **Code Name** | **OB (%)** | **DL** | **Drug** |
| --- | --- | --- | --- | --- | --- |
| MOL000273 | (2R)-2-[(3S,5R,10S,13R,14R,16R,17R)-3,16-dihydroxy-4,4,10,13,14-pentamethyl-2,3,5,6,12,15,16,17-octahydro-1H-cyclopenta[a]phenanthren-17-yl]-6-methylhept-5-enoic acid | FL1 | 30.93 | 0.81 | FL |
| MOL000283 | Ergosterol peroxide | FL5 | 40.36 | 0.81 | FL |
| MOL000287 | 3beta-Hydroxy-24-methylene-8-lanostene-21-oic acid | FL6 | 38.70 | 0.81 | FL |
| MOL000289 | pachymic acid | FL7 | 33.63 | 0.81 | FL |
| MOL001921 | Lactiflorin | CS3 | 49.12 | 0.80 | CS |
| MOL000275 | trametenolic acid | FL2 | 38.71 | 0.80 | FL |
| MOL001924 | paeoniflorin | CS4 | 53.87 | 0.79 | CS |
| MOL000211 | Mairin | MDP1 | 55.38 | 0.78 | MDP |
| MOL001323 | Sitosterol alpha1 | TR1 | 43.28 | 0.78 | TR |
| MOL007004 | Albiflorin | CS14 | 30.25 | 0.77 | CS |
| MOL000279 | Cerevisterol | FL3 | 37.96 | 0.77 | FL |
| MOL004355 | Spinasterol | CS9 | 42.98 | 0.76 | CS |
| MOL000449 | Stigmasterol | CS10 | 43.83 | 0.76 | CS |
| MOL000290 | Poricoic acid A | FL8 | 30.61 | 0.76 | FL |
| MOL002776 | Baicalin | CS6 | 40.12 | 0.75 | CS |
| MOL006999 | stigmast-7-en-3-ol | CS13 | 37.42 | 0.75 | CS |
| MOL000291 | Poricoic acid B | FL9 | 30.52 | 0.75 | FL |
| MOL000292 | poricoic acid C | FL10 | 38.15 | 0.75 | FL |
| MOL000359 | sitosterol | A1 | 36.91 | 0.75 | CS, GZ, MDP |
| MOL000358 | beta-sitosterol | B1 | 36.91 | 0.75 | CS, GZ, TR |
| MOL000296 | hederagenin | C1 | 36.91 | 0.75 | FL, TR |
| MOL000282 | ergosta-7,22E-dien-3beta-ol | FL4 | 43.51 | 0.72 | FL |
| MOL005043 | campest-5-en-3beta-ol | CS17 | 37.58 | 0.71 | CS |
| MOL000493 | campesterol | TR18 | 37.58 | 0.71 | TR |
| MOL001361 | GA87 | TR14 | 68.85 | 0.57 | TR |
| MOL001342 | GA121-isolactone | TR5 | 72.7 | 0.54 | TR |
| MOL001351 | Gibberellin A44 | TR8 | 101.61 | 0.54 | TR |
| MOL001355 | GA63 | TR11 | 65.54 | 0.54 | TR |
| MOL001329 | 2,3-didehydro GA77 | TR3 | 88.08 | 0.53 | TR |
| MOL001352 | GA54 | TR9 | 64.21 | 0.53 | TR |
| MOL001353 | GA60 | TR10 | 93.17 | 0.53 | TR |
| MOL001360 | GA77 | TR13 | 87.89 | 0.53 | TR |
| MOL001328 | 2,3-didehydro GA70 | TR2 | 63.29 | 0.5 | TR |
| MOL001358 | gibberellin 7 | TR12 | 73.8 | 0.5 | TR |
| MOL001348 | gibberellin 17 | TR6 | 94.64 | 0.49 | TR |
| MOL001349 | 4a-formyl-7alpha-hydroxy-1-methyl-8-methylidene-4aalpha,4bbeta-gibbane-1alpha,10beta-dicarboxylic acid | TR7 | 88.6 | 0.46 | TR |
| MOL001340 | GA120 | TR4 | 84.85 | 0.45 | TR |
| MOL001002 | ellagic acid | CS1 | 43.06 | 0.43 | CS |
| MOL001918 | paeoniflorgenone | CS2 | 87.59 | 0.37 | CS |
| MOL007016 | Paeoniflorigenone | CS15 | 65.33 | 0.37 | CS |
| MOL006992 | (2R,3R)-4-methoxyl-distylin | CS12 | 59.98 | 0.3 | CS |
| MOL007374 | 5-[[5-(4-methoxyphenyl)-2-furyl]methylene]barbituric acid | MDP6 | 43.44 | 0.3 | MDP |
| MOL001368 | 3-O-p-coumaroylquinic acid | TR15 | 37.63 | 0.29 | TR |
| MOL000098 | quercetin | MDP5 | 46.43 | 0.28 | MDP |
| MOL001736 | (-)-taxifolin | GZ1 | 60.51 | 0.27 | GZ |
| MOL004576 | taxifolin | GZ6 | 57.84 | 0.27 | GZ |
| MOL000073 | ent-Epicatechin | GZ5 | 48.96 | 0.24 | GZ |
| MOL000422 | kaempferol | MDP3 | 41.88 | 0.24 | MDP |
| MOL000492 | (+)-catechin | A2 | 54.83 | 0.24 | CS, GZ, MDP |
| MOL002714 | baicalein | CS5 | 33.52 | 0.21 | CS |
| MOL002883 | Ethyl oleate (NF) | CS16 | 32.4 | 0.19 | CS |

GZ=Guizhi, FL=Fuling, MDP=Mudanpi , CS=Chishao , TR= Taoren
