# Supplementary material for: Exploring the mechanisms of Guizhifuling pills in the treatment of coronary spastic angina based on network pharmacology combined with molecular docking
Source: Medicine (Baltimore). 2024 Jul 19;103(29):e39014. doi: 10.1097/MD.0000000000039014 (PMC11398759; doi:10.1097/MD.0000000000039014)
Supplement: Supplementary file 2 [file medi-103-e39014-s002.docx]

**Table S2** Specific information for core targets.

| Number | Uniprot ID | Gene name | Protein name | Degree |
| --- | --- | --- | --- | --- |
| 1 | P40763 | STAT3 | Signal transducer and activator of transcription 3 | 39 |
| 2 | P05231 | TP53 | Interleukin-6 | 38 |
| 3 | P04637 | IL6 | Cellular tumor antigen p53 | 38 |
| 4 | P31749 | AKT1 | RAC-alpha serine/threonine-protein kinase | 36 |
| 5 | P01584 | JUN | Interleukin-1 beta | 34 |
| 6 | P35222 | CTNNB1 | Catenin beta-1 | 34 |
| 7 | P05412 | IL1B | Transcription factor Jun | 34 |
| 8 | Q16665 | EGFR | Hypoxia-inducible factor 1-alpha | 33 |
| 9 | P01375 | TNF | Tumor necrosis factor | 33 |
| 10 | P00533 | HIF1A | Epidermal growth factor receptor | 33 |
| 11 | P12931 | SRC | Proto-oncogene tyrosine-protein kinase Src | 31 |
| 12 | P42574 | CASP3 | Caspase-3 | 31 |
| 13 | P10415 | BCL2 | Apoptosis regulator Bcl-2 | 31 |
| 14 | P01106 | MYC | Myc proto-oncogene protein | 30 |
| 15 | P14780 | MMP9 | Matrix metalloproteinase-9 | 29 |
| 16 | Q16644 | MAPK3 | MAP kinase-activated protein kinase 3 | 28 |
| 17 | P42224 | STAT1 | Signal transducer and activator of transcription 1-alpha/beta | 28 |
| 18 | P60484 | PTEN | Phosphatidylinositol 3,4,5-trisphosphate 3-phosphatase and dual-specificity protein phosphatase PTEN | 28 |
| 19 | P07900 | HSP90AA1 | Heat shock protein HSP 90-alpha | 27 |
| 20 | P01133 | EGF | Pro-epidermal growth factor | 27 |
| 21 | P35354 | PTGS2 | Prostaglandin G/H synthase 2 | 27 |
| 22 | P28482 | MAPK1 | Mitogen-activated protein kinase 1 | 26 |
| 23 | P24385 | CCND1 | G1/S-specific cyclin-D1 | 26 |
| 24 | P10145 | CXCL8 | Interleukin-8 | 25 |
| 25 | P37231 | PPARG | Peroxisome proliferator-activated receptor gamma | 24 |
| 26 | P22301 | IL10 | Interleukin-10 | 24 |
| 27 | P03372 | ESR1 | Estrogen receptor | 24 |
| 28 | O60674 | JAK2 | Tyrosine-protein kinase JAK2 | 23 |
| 29 | P01579 | IFNG | Interferon gamma | 22 |
| 30 | P09038 | FGF2 | Fibroblast growth factor 2 | 22 |
| 31 | P04626 | ERBB2 | Receptor tyrosine-protein kinase erbB-2 | 21 |
| 32 | Q96EB6 | SIRT1 | NAD-dependent protein deacetylase sirtuin-1 | 20 |
| 33 | P08253 | MMP2 | 72 kDa type IV collagenase | 20 |
| 34 | P49841 | GSK3B | Glycogen synthase kinase-3 beta | 18 |
| 35 | Q03135 | CAV1 | Caveolin-1 | 18 |
| 36 | P60568 | IL2 | Interleukin-2 | 17 |
| 37 | Q16236 | NFE2L2 | Nuclear factor erythroid 2-related factor 2 | 15 |
| 38 | P09601 | HMOX1 | Heme oxygenase 1 | 14 |
| 39 | P10721 | KIT | Mast/stem cell growth factor receptor Kit | 14 |
| 40 | P05067 | APP | Amyloid-beta precursor protein | 13 |
| 41 | P05121 | SERPINE1 | Plasminogen activator inhibitor 1 | 11 |
| 42 | P17252 | PRKCA | Protein kinase C alpha type | 10 |
| 43 | P00519 | ABL1 | Tyrosine-protein kinase ABL1 | 9 |
| 44 | P02741 | CRP | C-reactive protein | 9 |
