# Supplementary material for: Exploring the mechanisms of Guizhifuling pills in the treatment of coronary spastic angina based on network pharmacology combined with molecular docking
Source: Medicine (Baltimore). 2024 Jul 19;103(29):e39014. doi: 10.1097/MD.0000000000039014 (PMC11398759; doi:10.1097/MD.0000000000039014)
Supplement: Supplementary file 3 [file medi-103-e39014-s003.docx]

**Table S3** The top 20 KEGG pathways.

| **Entry ID** | **Description** | **GeneRatio** | **p.adjust** | **Count** |
| --- | --- | --- | --- | --- |
| hsa05200 | Pathways in cancer | 0.3082 | 5.37E-80 | 86 |
| hsa04151 | PI3K-Akt signaling pathway | 0.2043 | 3.98E-51 | 57 |
| hsa04933 | AGE-RAGE signaling pathway in diabetic complications | 0.1326 | 7.41E-48 | 37 |
| hsa04931 | Insulin resistance | 0.0860 | 1.05E-25 | 24 |
| hsa04020 | Calcium signaling pathway | 0.0968 | 3.80E-20 | 27 |
| hsa01524 | Platinum drug resistance | 0.0609 | 7.41E-19 | 17 |
| hsa04064 | NF-kappa B signaling pathway | 0.0681 | 7.41E-19 | 19 |
| hsa04630 | JAK-STAT signaling pathway | 0.0789 | 1.35E-18 | 22 |
| hsa04024 | cAMP signaling pathway | 0.0860 | 5.37E-18 | 24 |
| hsa04914 | Progesterone-mediated oocyte maturation | 0.0645 | 1.15E-17 | 18 |
| hsa05144 | Malaria | 0.0502 | 5.89E-17 | 14 |
| hsa04148 | Efferocytosis | 0.0717 | 9.77E-17 | 20 |
| hsa05202 | Transcriptional misregulation in cancer | 0.0753 | 4.57E-16 | 21 |
| hsa04611 | Platelet activation | 0.0609 | 7.24E-15 | 17 |
| hsa04371 | Apelin signaling pathway | 0.0609 | 4.90E-14 | 17 |
| hsa04152 | AMPK signaling pathway | 0.0573 | 8.32E-14 | 16 |
| hsa04110 | Cell cycle | 0.0609 | 3.55E-13 | 17 |
| hsa04217 | Necroptosis | 0.0609 | 4.27E-13 | 17 |
| hsa04520 | Adherens junction | 0.0466 | 9.55E-12 | 13 |
| hsa04750 | Inflammatory mediator regulation of TRP channels | 0.0466 | 1.86E-11 | 13 |
